# La myosite ossifiante circonscrite, une localisation inhabituelle - à propos d'un cas et revue de la littérature

**DOI:** 10.11604/pamj.2016.24.71.9043

**Published:** 2016-05-20

**Authors:** Ikram Taam, Khouloud Boussouni, Bouchaib Redouane, Touriya Amil, Rachida Saouab

**Affiliations:** 1 Service d'Imagerie Médicale, Hôpital Militaire d'Instruction Mohammed V, Rabat, Maroc

**Keywords:** myosite ossifiante, paravertébral, IRM, Myositis ossificans, paravertebral, MRI

## Abstract

La myosite ossifiante circonscrite (MOC) est une pathologie rare caractérisée par une ossification hétérotopique non tumorale des tissus mous. C'est une pathologie du sujet jeune, survenant le plus souvent après un traumatisme. Sa localisation est ubiquitaire, prédominante au niveau des ceintures et des membres. Nous rapportons l'observation d'une jeune patiente présentant une MOC paravertébrale sans contexte traumatique, dans le but de rappeler les critères diagnostics et les aspects en imagerie.

## Abstract (en)

Myositis ossificans circumscripta (MOC) is a rare condition characterized by nontumoral heterotopic ossification of the soft tissues. This condition affects young subjects, occurring mainly after trauma. It is ubiquitous, predominantly located in girdles and limbs. We report the case of a young patient with paravertebral MOC without traumatic context; the aim of this study was to recall diagnostic criteria and imaging aspects.

## Introduction

La myosite ossifiante circonscrite (MOC) est une prolifération hétérotopique non tumorale du tissu osseux et cartilagineux à partir du tissu conjonctif interstitiel au sein des parties molles du squelette, et présente 0.7% des pseudotumeurs des parties molles. Elle survient chez le sujet jeune, souvent à la suite d'un traumatisme, et siège principalement au niveau des ceintures pelviennes, scapulaires, la cuisse et le bras. Les données clinico-biologiques ne sont pas spécifiques, d'où l'intérêt de l'imagerie médicale qui est primordiale pour le diagnostic positif et le suivi. Le diagnostic est parfois confirmé par l'histologie. Nous rapportons un nouveau cas de myosite ossifiante circonscrite non traumatique avec une localisation inhabituelle chez une jeune patiente.

## Patient et observation

**Figure 1:**
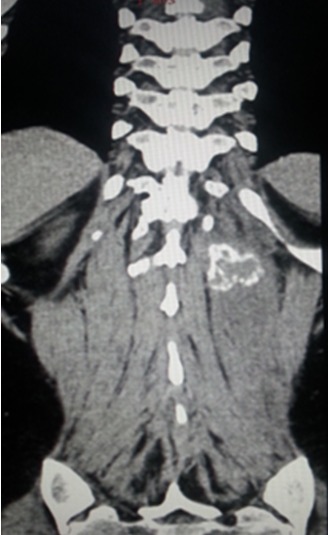
Coupe scannographique coronale du rachis dorsolombaire montrant une masse hypodense bien limitée avec une coque périphérique calcifiée au niveau du muscle paravertébrale gauche qui parait tuméfié

**Figure 2:**
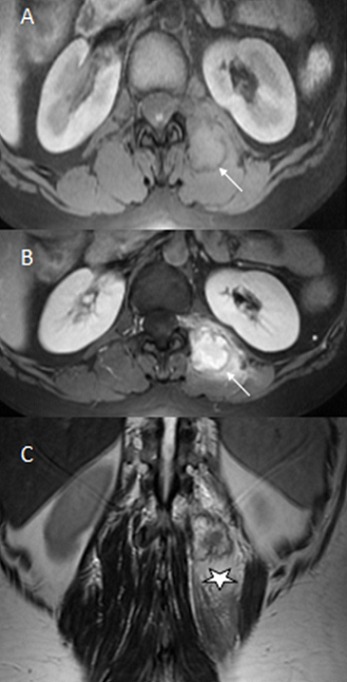
IRM lombaire en séquence pondéré T1 (A), T2 (B) et après injection de Gado (C): masse du muscle paravértébral gauche en isosignal T1, hypersignal hétérogène T2, entourée d'un halo en hyposignal T1 et T2 (calcifications) (flèche), se rehaussant de façon hétérogène après injection, avec présence d'un liseré de séparation entre la masse et la corticale adjacente, le muscle érecteur du rachis parait en hyposignal T1 et hypersignal T2 se rehaussant de façon intense après injection de produit de contraste témoignant de son inflammation (étoile)

Patiente âgée de 37 ans, sans antécédent pathologiques particuliers, notamment pas de traumatisme récent, qui s'est présenté en consultation neurochirurgicale pour des dorsolombalgies de type inflammatoire, d'installation rapidement progressive sur trois semaines. L'examen clinique objectivait une douleur paravértébrale gauche vive à la palpation au niveau de la charnière dorsolombaire sans signes inflammatoires en regard. Le bilan biologique révélait un syndrome inflammatoire sans syndrome infectieux. La tomodensitométrie dorsolombaire mettait en évidence une masse grossièrement ovalaire, bien limitée au dépend du muscle érecteur du rachis gauche, en regard de L1 et L2 discrètement hypodense par rapport au muscle, mesurant 85x35x25 mm avec des calcifications périphériques, on notait l'aspect tuméfié et infiltré du muscle érecteur du rachis gauche par rapport au muscle controlatéral (Figure 1), le diagnostic de myosite ossifiante circonscrite a été suspecté. L'imagerie par résonnance magnétique confirmait la présence d'une masse du muscle érecteur du rachis gauche en isosignal T1, hypersignal hétérogène T2, entourée d'un halo en hyposignal T1 et T2 (calcifications), se rehaussant de façon hétérogène après injection de gadolinium, avec présence d'un liseré de séparation entre la masse et la corticale adjacente, le muscle érecteur du rachis parait en hyposignal T1 et hypersignal T2 se rehaussant de façon intense après injection de produit de contraste témoignant de son inflammation (Figure 2) Sur les données fournies par la TDM et L'IRM le diagnostic de myosite ossifiante circonscrite a été retenu. La patiente a été mise sous traitement anti-inflammatoire, et elle a été suivie pendant 6 mois avec bonne évolution clinique et biologique.

## Discussion

La myosite ossifiante est une prolifération hétérotopique non néoplasique d'os et de cartilage au sein des structures musculaires. C'est une lésion bénigne, rare, représentant 0.7% des pseudotumeurs des parties molles [1], il existe deux formes de myosite ayant des caractéristiques histologiques proches mais des présentations cliniques différentes, il s'agit de la myosite ossifiante circonscrite et la myosite ossifiante progressive généralisée d'ordre génétique ou myosite de Munchmeyer [2]. Le terme de myosite est cependant inapproprié car l'ossification ne provient pas des fibres musculaires striées squelettiques mais du tissu conjonctif interstitiel [3]. La myosite ossifiante circonscrite (MOC) est souvent rattachée à un traumatisme (60%-75 des cas) [4] ou à des microtraumatismes répétés. Cela est expliqué par la réponse à une hémorragie ou nécrose tissulaire post-traumatique par un phénomène de réparation avec réaction fibroblastique et métaplasie osseuse et cartilagineuse. Cependant il n'est pas toujours possible de la rattacher à un traumatisme connu, il s'agit de formes apparemment spontanées, appelées myosite ossifiante circonscrite non traumatique [2]. L'absence de notion de traumatisme a été rapportée par notre patiente. Sur le plan histologique La myosite ossifiante se caractérise par trois phases: Au cours de la première semaine: une prolifération active mésenchymateuse de fibroblastes. À la deuxième semaine: apparition d'ostéoblastes hétérotopiques avec production d'une matrice ostéoïde et création d'une capsule fibreuse. Dans les deux à huit semaines suivantes, constitution d'un « phénomène de zone», avec trois zones distinctes: zone centrale (tissu mésenchymateux, mitoses, fibroblastes, hémorragies, nécrose), zone intermédiaire (ostéoblastes, ilots d'os immature), zone externe (des travées d'os mature). L'ossification se fait de façon centripète [5]. Le site lésionnel de prédilection est périphérique et généralement proximal: les ceintures pelviennes, scapulaires, la cuisse, bras, parfois distal: au niveau des mains et des pieds. Elle intéresse le compartiment antérieur plus que le compartiment postérieur [6]. L'atteinte des muscles paravértebraux est très rarement décrite [7]. La MOC survient chez le sujet adulte jeune et se manifeste par l'apparition brutale d'une masse douloureuse des parties molles, de volume d'emblée maximal accompagné de signes inflammatoires cliniques et biologiques à la phase initiale et qui diminuent puis se normalisent durant la phase subaigüe et la phase chronique [5]. L'aspect radiologique change parallèlement avec la maturation histologique de la lésion [8].

**A la phase aigue:** Les radiographies standards sont normales ou montrent une tuméfaction des parties molles avec réaction périostée de l'os sous jacent sans calcification. L’échographie met en évidence des anomalies de l’échogénicité du muscle et de vascularisation mais sans aucune spécificité [9, 10]. La TDM objective à ce stade une tuméfaction des parties avec un processus lésionnel mallimité, se rehaussant après injection de produit de contraste sans image de calcification avec respect de l'os adjacent. En IRM, la masse apparait en isosignal T1, en hypersignal T2 hétérogène, faiblement rehaussée après injection de gadolinium, avec un œdème musculaire extensif dépassant les limites delà tuméfaction [11] Cet aspect œdémateux des muscles peut faire évoquer également un abcès, un hématome ou la rabdomyolyse d'où la nécessité d'une confrontation clinico-biologique et radiologique [12].

**A la phase intermédiaire:** Les radiographies standards et la TDM objectivent la présence de calcifications floconneuse évoluant en ossifications périphérique (couronne), l'ossification est séparée de l'os par une bande radiotransparente (liseré de sécurité). L’échographie montre le phénomène des trois zones: la zone périphérique hypoéchogène correspondant à l′hyperémie et l’œdème péri-lésionnelle. La deuxième zone hyperéchogène en raison de la calcification et la zone la plus interne est hypoéchogène correspondant au contenu de la masse. En IRM, la masse est en hyposignal T1 et hypersignal T1 et T2 au centre avec un halo en hyposignal T1 et T2 ( calcifications), parfois un aspect de niveau liquide-liquide intralésionnel peut être observé [13], durant cette période, les principaux diagnostics différentiels sont les rhabdomyosarcomes, les synovialosarcomes dont les calcifications sont centrales et dispersées avec atteinte de l'os adjacent alors que dans la myosite ossifiante circonscrite les calcifications sont périphériques et continues avec respect de l'os), les tendinites calcifiantes peuvent être évoquées mais elles se caractérisent par des calcifications sans aucune masse bien limitée [13]. Phase chronique: Les radiographies standards et la TDM montrent la maturation de l'ossification à la périphérie de la masse parfois une masse totalement calcifiée; et cette ossification peut arriver au contact de l'os, en IRM, les calcifications se présentant sous forme de structure osseuse avec sa corticale et sa graisse centrale autour parfois d'un nodule fibreux sans œdème périlésionnel [13]. Durant cette phase, le principal diagnostic différentiel est l'ostéosarcome paraostéal qui est caractérisé par une ossification qui se fait de façon centrifuge, avec un centre plus dense contrairement à la myosite ossifiante circonscrite dont l'ossification se fait de façon centripète [4]. La scintigraphie objective l'hyperfixation intense dans les tissus mous durant les phases aigüe et subaigüe, et diminue avec le temps (phase chronique) [5]. En cas de doute diagnostique, un contrôle par l'imagerie à 15jours peut permettre d’éviter une biopsie chirurgicale aux patients, la biopsie n'est indiquée que quand l'aspect évolutif de la lésion et l'aspect radiologique sont atypiques, pour éliminer un processus malin. L'examen histologique peut être trompeur, il doit être fait sur un volume suffisant de la masse pour retrouver le phénomène de zone (tissu mésenchymateux - ostéoblastes et îlots d'os immature - travées d'os mature), si non on peut porter le diagnostic de tumeur maligne [9–14]. L’évolution de la myosite ossifiante circonscrite se fait vers la stabilisation [15], la régression ou la disparition de la lésion, la dégénérescence sarcomateuse semble exceptionnelle lorsque le diagnostic initial de MOC est avéré [1]. L'abstention thérapeutique est la règle et c'est le cas de notre patiente. La chirurgie n'est indiquée que dans les cas des complications neurologiques ou quand elle touche l'insertion d'un muscle ou un tendon et perturbe leur fonction [16].

## Conclusion

La myosite ossifiante circonscrite (MOC) est une entité pathologique à connaitre. L'imagerie reste primordiale pour le diagnostic. Elle repose essentiellement sur les radiographies standard et la TDM qui mettent en évidence des calcifications centripètes dans les parties molles. L'IRM a intérêt dans le diagnostic précoce mais reste non spécifique.
